# Tumoral indoleamine 2, 3-dioxygenase 1 is regulated by monocytes and T lymphocytes collaboration in hepatocellular carcinoma

**DOI:** 10.18632/oncotarget.7438

**Published:** 2016-02-17

**Authors:** Qiyi Zhao, Pei-pei Wang, Zhan-lian Huang, Liang Peng, Chaoshuang Lin, Zhiliang Gao, Shicheng Su

**Affiliations:** ^1^ Department of Infectious Diseases, Third Affiliated Hospital, Sun Yat-sen University, Guangzhou, China; ^2^ Key Laboratory of Gene Engineering of the Ministry of Education, State Key Laboratory of Biocontrol, School of Life Sciences, Sun Yat-sen University, Guangzhou, China; ^3^ Guangdong Provincial Key Laboratory of Liver Disease, The Third Affiliated Hospital of Sun Yat-sen University, Guangzhou, China; ^4^ Guangdong Provincial Key Laboratory of Malignant Tumor Epigenetics and Gene Regulation, Medical Research Center, Sun Yat-Sen Memorial Hospital, Sun Yat-Sen University, Guangzhou, China; ^5^ Breast Tumor Center, Sun Yat-Sen Memorial Hospital, Sun Yat-Sen University, Guangzhou, China

**Keywords:** IDO1, tumor cells, monocytes, T cells

## Abstract

Indoleamine 2, 3-Dioxygenase 1 (IDO1) in cancer cells plays a critical role in tumor immunosuppression. However, the precise mechanisms regulating tumoral IDO1 expression in tumor milieus remain unclear. Here, we reported that IDO1 expression in tumor cells of hepatocelluar carcinomas (HCC), displayed a discrete rather than uniform pattern. *In vitro* culture, human hepatoma cell lines did not constitutively express IDO1. Interestingly, co-culture with peripheral blood mononuclear cells (PBMC) significantly induced and maintained IDO1 expression in these tumor cells, predominantly through IFN-γ. Mechanistically, we showed that IDO1 expression in tumor cells was only induced when co-cultured with both T lymphocytes and monocytes. Moreover, the cooperation between T lymphocytes and monocytes played an indispensable role on the tumoral IDO1 expression in immunocompromised mice. Taken together, our data supported the notion that IDO1 expression in tumor cells might serve as a counter-regulatory mechanism regulated by immune system, and provided new insights into the collaborative action of different inflammatory cells in tumor immunosuppression.

## INTRODUCTION

Indoleamine 2, 3-Dioxygenase 1 (IDO1) expression has been found to be up-regulated and associated with poor prognosis in various types of human cancers [1–4]. In human cancer samples, IDO1 is expressed in the tumor cells, as well as in stromal cells such as monocytes/macrophages, dendritic cells (DCs), endothelial cells or fibroblasts in response to the tumor [1, 5]. Tumoral IDO1 expression plays an indispensable role in local immune suppression within the tumor microenvironment [6, 7]. It has been reported that up-regulation of IDO1 in metastatic malignant melanoma cells and metastatic pancreatic ductal adenocarcinoma cells was associated with an increased number of regulatory T cells (Tregs) and shorter survival [8, 9]. By inhibiting the tumoricidal effects of T cells, IDO1-high expression by colorectal tumor cells significantly contributes to disease progression and poor overall survival in patients with colorectal cancer [10]. In addition, IDO1 in serous ovarian cancer cells could serve as a marker of poor prognosis and is positively associated with paclitaxel resistance [11].

Despite the expression and immune-suppressive role of IDO1 have been well documented in numerous studies, one unclear question is how IDO1 in human tumor cells is regulated by tumor milieus [4]. Varying degrees of tumoral IDO1 expression has been described in biopsy material in a variety of human cancers [6, 12]. However, experimentally, no detectable IDO1 expression is observed in most human tumor cell lines oriented from pancreas, cervix, colon, melanoma, possibly due to the absence of a complete cancer microenvironment *in vitro* [8-10, 13, 14]. However, those tumor cell lines without constitutive IDO1 expression could be invariably induced by certain stimuli such as IFN-γ [8-10, 13, 14], which is presumably analogous to actual state *in vivo*. Consistently, studies in mouse tumor model have reported that up-regulation of immunosuppressive molecules such as IDO1 and B7-H1 in the melanoma cells is driven by CD8^+^ T cells [15]. In addition, the genetic and epigenetic alterations in cancer cells could license their response to the tumor milieu [16–19]. In that regard, genetic studies in mice demonstrated that Bin1 mutations could potentiate IFN-γ-induced NFκB and STAT-dependant IDO1 overexpression in tumor cells [20].

Here, we explore a mechanism for tumoral IDO1 expression regulated by immune system in human hepatocelluar carcinomas (HCC). We examined the distribution pattern of tumoral IDO1 in 64 human HCC samples. Discrete tumoral IDO1 expression was observed in most of the IDO1-positive samples, with abundant immune cells infiltration. By *in vitro* and *in vivo* studies, we showed that cooperation of T lymphocytes and monocytes modulated the tumoral IDO1 expression, which was driven by pro-inflammatory cytokines. These data indicate an adaptive mechanism-driven expression of IDO1 in tumor cells.

## RESULTS

### Discrete tumoral IDO1 expression in cancer patients

**Figure 1:**
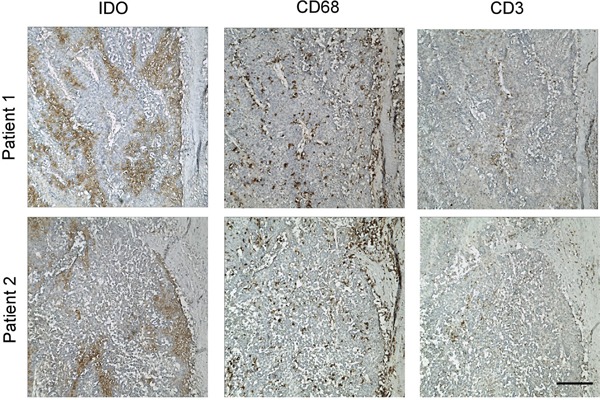
Discontinuous tumoral IDO1 expression in human hepatoma samples Paraffin-embedded hepatocelluar carcinoma samples were stained for Ab against IDO1. Positive signals appear in brown (DAB staining). One of five representative areas is shown. Scale bar, 200 μm.

To investigate the expression pattern of IDO1 in various types of human cancers, we performed immunohistochemistry staining for IDO1 in samples of hepatocelluar carcinomas (n = 64). IDO1-positive cancer cells were detected in nearly half of the tumor samples analyzed (47%, 30 of 64). Interestingly, intracellular IDO1 expression in tumor cells displayed a regional rather than uniform expressing pattern, which often occurred in discrete geographic foci (Figure 1). Although some cancerous cells were strongly positive for IDO1, the staining of others in adjacent areas was much weaker or negative (Figure 1). We grouped the tumors into 3 categories according to the proportion of tumor cells positive for IDO1. Among 30 tumoral IDO1^+^ positive samples, 22 (73%) contained less than 10% IDO1-positive tumor cells, 8 (26%) contained 10-50% IDO1-positive tumor cells, while few with more than 50% positive. Such a discrete tumoral IDO1 expression pattern was dominant in hepatocelluar carcinomas, with abundant immune cells infiltration in the same area (Figure 1), suggesting that IDO1 expression in these tumors are likely regulated by tumor milieus.

### Immune cells were required for the sustained IDO1 expression in human hepatoma cell lines

**Figure 2:**
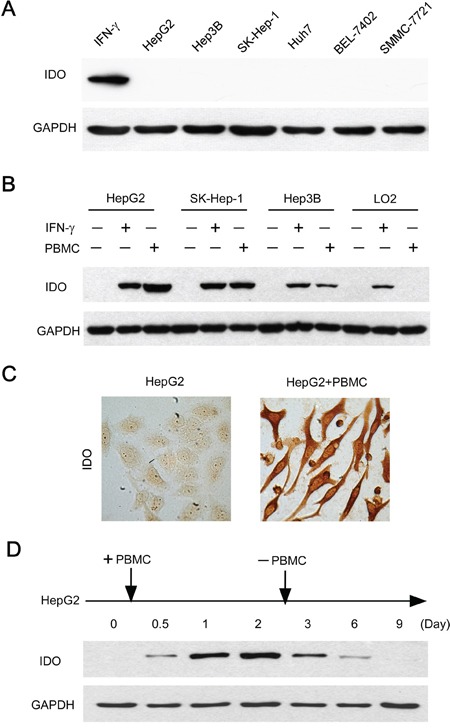
Immune cells contributed to the induction and maintenance of IDO1 in human hepatoma cells **A.** Human hepatoma (HepG2, SK-Hep-1, Hep3B, Huh7, SMMC-7721 and BEL-7402) cell lines did not constitutively express IDO1 *in vitro*. The IFN-γ treated HepG2 served as a positive control. **B.** Tumor cells (HepG2, SK-Hep-1, Hep3B) or normal liver cells (L02) were cultured with PBMC at a ratio of 1: 3 or IFN-γ (100 IU/ml) as IDO1-positive control for 2 days. For the coculture groups, PBMC were wash away before protein extraction. The expression of IDO1 protein in the attached cells was detected by western blot. **C.** HepG2 were cultured alone or with PBMC for 2 days. IDO1 protein was detected by immunocytochemistry. One of five representative areas is shown. **D.** PBMC were added to HepG2 at day 0, and were washed away after 2-day coculture. The IDO1 protein in the attached cells was detected by western blot at indicated times.

We then investigated whether human tumor cell lines oriented from hepatocelluar carcinomas constitutively expressed IDO1 *in vitro*. Unexpectedly, IDO1 expression were not detectable in all of the 6 human hepatoma cell lines grown *in vitro* (Figure 2A), suggesting that additional factors within the tumor milieu are required for inducing IDO1 in tumor cells. Recent studies have reported the contribution of immune cells in the up-regulation of IDO1 in mouse melanoma model [15]. However, whether immune cells could influence the IDO1 in human hepatoma cells remains unclear. We therefore investigated it in a co-culture model *in vitro*: 3 tumor cell lines without constitutive IDO1 expression, including HepG2, SK-Hep-1 and Hep3B were co-cultured with peripheral blood mononuclear cells (PBMC). Strikingly, all tumor cells showed significant IDO1 expression by western blot after a 2-day co-culture with PBMC, at levels comparable to those induced by IFN-γ (Figure 2B). The intracellular distribution of IDO1 protein within tumor cells was confirmed by immunocytochemistry (Figure 2C). In contrast, normal liver cells (L02) showed no IDO1 expression when cocultured with PBMC; although its IDO1 could be induced by a positive stimulator IFN-γ (Figure 2B). These data suggested that PBMC can induce the expression of IDO1 in hepatoma cells.

We then ask whether PBMC were necessary for the maintenance of IDO1 expression in tumor cells. Measuring IDO1 protein by HepG2 cells co-cultured with PBMC over time revealed an increasing level of IDO1 expression, reaching a maximum within 2 days (Figure 2D). Surprisingly, when PBMC were removed from the co-culture systems at day 2, the IDO1 expression by HepG2 cells gradually declined from day 3, then returned to a basal level after 9 days (Figure 2D). Collectively, these data revealed that immune cells are required for the induction and maintenance of IDO1 protein in those human hepatoma cells without constitutive IDO1 expression.

### Induction of tumoral IDO1 is pro-inflammatory cytokines driven

**Figure 3:**
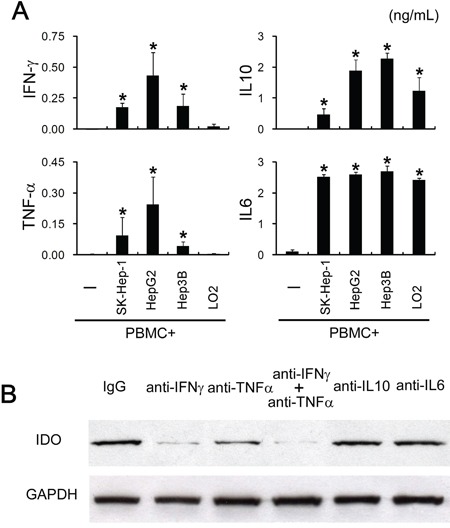
Tumoral IDO1 expression was regulated by pro-inflammatory cytokines **A.** PBMC were cocultured with tumor cells lines (HepG2, SK-Hep-1, Hep3B) or normal liver cells (L02), and the production of cytokine (TNF-α, IFN-γ, IL-6, IL-10) was measured by ELISA. **B.** HepG2 cocultured with PBMC were treated with indicated blocking Abs or control IgG. The expression of IDO1 in HepG2 was determined by western blot and ELISA.

Expression of IDO1 could be induced or maintained by different inflammatory cytokines depending on specific cell types [21–23]. To study the mechanisms involved in the tumoral IDO1 induction by immune cells, we first investigated the cytokines production in the co-culture system. Measuring inflammatory cytokines levels in co-culture systems revealed a moderate accumulation of IFN-γ and TNF-α, as well as a substantial amount of IL-10 and IL-6 (Figure 3A). mRNAs of these cytokines were not detectable in the tumor cells by quantitative RT-PCR (data not shown), suggesting that these inflammatory cytokines were secreted by PBMC in the cocultures. To determine which cytokine(s) is responsible for the tumoral IDO1 expression, we used specific neutralizing antibodies to abolish the effects of IFN-γ, TNF-α, IL-10 or IL-6. Blocking IFN-γ effectively inhibited the up-regulation of IDO1 protein in HepG2 cells cocultured with PBMC (Figure 3B). Interestingly, neutralizing TNF-α had a synergy effect, whereas it was not affected by treatment with a concentration of anti-IL-10 or anti-IL-6 Ab that effectively neutralized them in the cocultures (Figure 3B). In contrast, normal liver cells (L02) which showed no IDO1 expression when cocultured with PBMC (Figure 2A), failed to trigger IFN-γ and TNF-α production in the coculture system, although comparable level IL-10 and IL-6 could be detected (Figure 3A). These data indicated that the elevation of tumoral IDO1 by immune cells is predominantly driven by IFN-γ, and that TNF-α could potentiate the IFN-γ–mediated IDO1 expression.

### Cooperation of T lymphocytes and monocytes modulates the tumoral IDO1 expression

**Figure 4:**
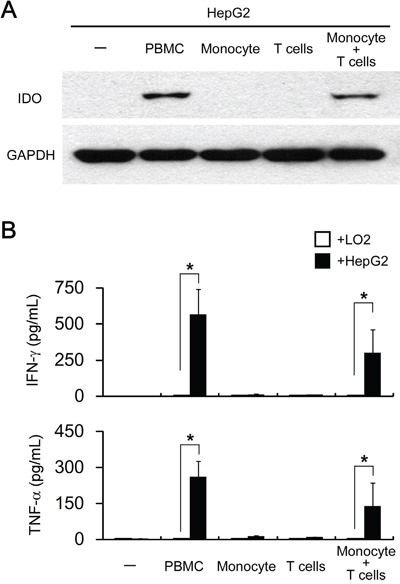
Both T lymphocytes and monocytes are responsible for pro-inflammatory cytokines driven tumoral IDO1 expression *in vitro* HepG2 (hepatoma cell line) or L02 (normal liver cell line) were cocultured with PBMC (1: 3) or with purified monocytes or/and T cells (1: 1: 2) for 2 days. **A.** The expression of IDO1 in HepG2 cells were determined by western blot. **B.** The production of IFN-γ and TNF-α was determined by ELISA. *p < 0.05 are shown.

Next, we try to determine which cellular component(s) in PBMC contribute to the pro-inflammatory cytokines driven tumoral IDO1 expression. Activated T cells have been reported to relate to the IDO1 expression in mouse tumors [15]. We therefore purified T lymphocytes from PBMC by negative magnetic sorting (untouched T cells), and cocultured them with HepG2. Unexpectedly, such treatments failed to induce IDO1 expression in HepG2 or IFN-γ/TNF-α production in co-culture supernatant (Figure 4A), suggesting that additional components in PBMC are required for the T cells activation and tumoral IDO1 induction.

We recently observed that tumor-associated macrophages (TAM) could effectively activate T cells to express CD69 and produce a certain amount of IFN-γ [24]. To investigate whether such a mechanism is involved in tumoral IDO1 expression, we co-cultured HepG2 with T lymphocytes plus monocytes. As expect, HepG2 co-cultured with T lymphocytes and monocytes up-regulated IDO1 protein at levels comparable to those cocultured with PBMC (Figure 4A). In consistent with IDO1 level, production of IFN-γ and TNF-α were detected in the coculture of T lymphocytes and monocytes in the present of HepG2, but not in co-culture of monocytes and HepG2, or coculture of T lymphocytes and monocytes (Figure 4B and data not shown). Collectively, our data indicated that cooperation of T lymphocytes and monocytes are indispensable for the up-regulation of IDO1 in tumor cells *in vitro*.

### T lymphocytes and monocytes contribute to IDO1 elevation *in vivo*

**Figure 5:**
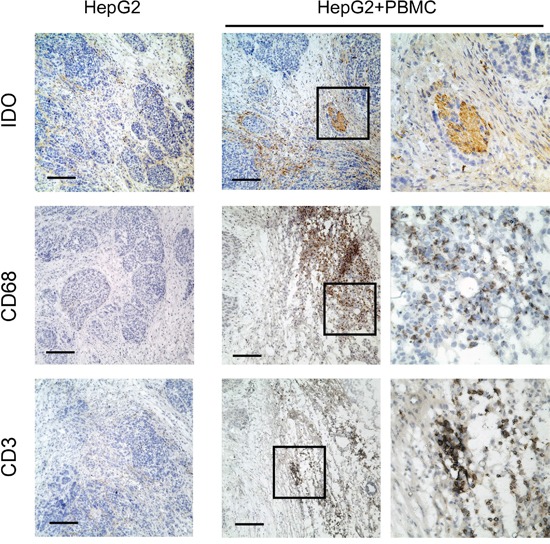
Human T lymphocytes and monocytes elevated IDO1 in human tumor cells in a xenograft model *in vivo* Human hepatoma (HepG2) was implanted subcutaneously into nonobese diabetic/severe combined immunodeficient (NOD/SCID) mice alone, or co-implantation with human PBMC (1:3) as described in materials and methods. Paraffin-embedded tumor tissues were stained for Abs against human IDO1, CD68 or CD3, respectively. Scale bar, 100 μm.

As the *in vitro* data suggested that monocytes and T lymphocytes cooperated to induce IDO1 expression in tumor cells, we further confirm our finding *in vivo*. Human hepatoma cells HepG2, which did not express IDO1 *in vitro*, were subcutaneously implanted into non-obese diabetic/severe combined immunocompromised (NOD/SCID) mice (Figure 4A and Figure 5). As expected, IDO1 expression was undetectable in xenografts when tumor cells were inoculated alone (Figure 5). In contrast, co-implantation of HepG2 cells with human PBMC into NOD-SCID mice revealed abundant T lymphocytes and monocytes infiltration in xenografts (Figure 5). More importantly, strong IDO1 expression in tumor cells was observed in the xenograft of co-implantation group (Figure 5). Collectively, our data revealed that T lymphocytes and monocytes play an indispensible role on IDO1 induction in tumor cells *in vivo*.

## DISCUSSION

IDO1 has been recognized as an important mediator for tumor immune suppression, but it precise regulating mechanisms in human cancer cells are not fully understood [4, 21, 22, 25]. Here, we observed that discrete tumoral IDO1 expression pattern was dominant in patients with hepatocelluar carcinomas. Mechanistically, immune cells were required for the induction and maintenance of IDO1 expression in human tumor cells. Furthermore, we showed that monocytes cooperated with T lymphocytes to modulate the inflammatory cytokines-driven tumoral IDO1 elevation.

How IDO1 expression in human tumor cells is regulated by tumor milieus remain unclear [4]. Our present study showed constitutive expression of IDO1 in tumor cells of various cancer types is rare, and cooperation of T cells and monocytes induce tumoral IDO1 expression through inflammatory cytokine. This conclusion is based on the following evidence. First, 6 human hepatoma cell lines we examined did not constitutively express IDO1. However, their IDO1 expressions were strongly induced when co-cultured with PBMC. Furthermore, such IDO1 expression gradually declined and finally returned to the basal level when PBMC were removed. Second, TNF-α had a synergy effect with IFN-γ in the immune cells mediated up-regulation of tumoral IDO1. Third, by *in vitro* and *in vivo* studies, we showed that cooperation of T lymphocytes and monocytes contributed to the induction of tumoral IDO1 expression. In accordance with the above findings, both our data (Figure 1) and other studies showed tumoral IDO1 expression in clinical samples of various cancer types displayed a discrete but not uniform expression pattern [9–12]. We (Figure 1) and others observed that the tumoral IDO1 expression was most pronounced usually at the invasive front, where inflammatory cells predominantly located [6, 12]. Exceptionally, IDO1-expressing tumor cells were often found in the absence of any inflammation in endometrial carcinomas [4, 12]. Of note, IDO1 activities in female reproductive tract intrinsically exist to provide an immunosuppressive barrier that protects allogeneic conceptuses from maternal T-cell immunity [26]. Taken together, these observations supported that the induction and maintenance of IDO1 in tumor cells are regulated by tumor-infiltrating inflammatory cells. Such a pattern was dominant, at least in case in tumors associated with inflammation.

Emerging evidence has shown the interrelationship between inflammation and induced immunosuppression in cancer, within which different mediators contribute to inflammation-associated immunosuppression [27–32]. Interestingly, several studies suggested that immunosuppressive cells or mediators may serve as counter-regulatory mechanisms, which are induced by inflammatory response in tumor [15, 33]. For example, the recruitment of Tregs to melanoma was depended on the presence of CD8^+^ T cells [15, 33]. In melanoma and HCC, T cells might actually trigger their own inhibition by secreting IFN-γ or IL-17 that drove PD-L1 expression [33, 34]. Extending the current knowledge on cancer-related inflammation, our present study suggested an adaptive tumor immune escape mechanism, which is overexpression of immunosuppressive IDO1 under the stimuli of IFN-γ derived from immune cells.

Previous studies have reported the contribution of T cells in inducing IDO1 in tumor cells. In these studies, either anti-CD3/CD28 or high level of IL-2 was used to activated T cells to generate IFN-γ, which is responsible for the IDO1 induction in their system [13, 35, 36]. However, such external anti-CD3/CD28 or IL-2 models are artificial and do not reflect the actual state in tumor microenvironment. Our present study showed that tumor cells cocultured with purified, untouched T cells failed to elevate IDO1 expression. In contrast, tumoral IDO1 expression was induced when tumor cells were cocultured with PBMC or a mixture of monocytes and T cells. These studies demonstrate that not only T cells, but also other inflammatory cells are required for sufficient tumoral IDO1 induction.

In summary, our study provides important new insights into the collaborative action of immune cells that is exercised to up-regulate immunosuppressive IDO1 in hepatoma cells. These data support the notion that IDO1 in tumor cells might serve as a counter-regulatory mechanism stimulated by inflammatory response. Further studies are warrant to investigate clinical benefit of cancer immunotherapy targeting IDO1 in tumor patients.

## MATERIALS AND METHODS

### Patients and specimens

Tumor tissues samples were obtained from the Third Affiliated Hospital of Sun Yat-sen University. 64 patients with hepatocellular carcinomas underwent curative resection between 2009 and 2013, and samples from these patients were used for immunohistochemistry. All samples were anonymously coded in accordance with local ethical guidelines (as stipulated by the Declaration of Helsinki), and written informed consent was obtained. The protocol was approved by the Review Board of Third Affiliated Hospital of Sun Yat-sen University.

### Abs and reagents

The Abs and chemicals used and their sources were as follows: mouse anti-human IDO (clone: 10.1) from Chemicon/Merck Millipore; the blocking mAbs against human TNF-α (clone: 28401), IFN-γ (clone: 25723), IL-6 (clone: 6708), IL-10 (clone: 23738) and control IgG from R&D Systems; Envision System for immunohistochemistry from Dako. All other reagents were obtained from Sigma-Aldrich unless otherwise indicated in the text.

### Cell lines and cultures

Human hepatomacell lines HepG2, SK-Hep-1, Hep3B were obtained from the American Type Culture Collection; human normal liver cells (L02), hepatoma (Huh7, SMMC-7721, BEL-7402) cell lines were from the Cell Bank, Chinese Academy of Sciences. All cells were tested for mycoplasma contamination using a single-step PCR method, and they were maintained in DMEM supplemented with 10% FBS [37]. HepG2 incubated with IFN-γ (100 IU/ml) for 2 days were served as IDO1-positive control.

### Isolation of PBMC and T cells from peripheral blood

Peripheral blood mononuclear cells (PBMC) were isolated from buffy coats derived from the blood of healthy donors by Ficoll density gradient centrifugation as described previously [37]. Monocytes or T lymphocytes were purified from PBMCs by using anti-CD14 magnetic beads or Pan T Cell Isolation Kit II respectively according to the manufacturer's instructions (MiltenyiBiotec).

### *In vitro* coculture model

Tumor or normal cell lines were cultured with PBMC (cell lines: PBMC = 1: 3), or with purified monocytes or/and T cells (cell lines: monocytes: T cells = 1: 1: 2) in RPMI1640 (eBioscience) supplemented with 10% FBS for indicated time. The medium were changed every other day. In some experiments, cells were pretreated with a specific blocking monoclonal Ab (5 μg/ml) against TNF-α, IFN-γ, IL-6, IL-10 or with a control Ab.

### *In vivo* tumor assay in a xenograft model

Animal protocols were approved by the Review Board of Sun Yat-Sen University. 10^6^ human hepatoma cells (HepG2) in 100 μl of buffered saline were subcutaneously injected into the dorsal tissues of NOD/SCID mice (5–7 weeks old) as our previous studies described [38]. In the HepG2+PBMC group, 3 × 10^6^ human PBMC were subsequently injected into dorsal tissues around the tumors in 100 μl of buffered saline on day 5 after HepG2 inoculation. After another 7 days, mice were sacrificed, and tumors were formalin-fixed and Paraffin-embedded. Immunohistochemistry was performed with antibodies specific for human IDO1, CD68 (DAKO), and CD3 (DAKO), and then analyzed as below.

### ELISA

Concentrations of TNF-α, IFN-γ, IL-6 and IL-10 in the conditioned media were detected by ELISA kits according to the manufacturer's instructions (eBioscience).

### Immunohistochemistry and immunocytochemistry

Paraffin-embedded samples were cut into 5-μm sections and processed for immunohistochemistry as previously described [39, 40]. Following incubation with the Ab against human IDO1, CD68 or CD3, the sections were stained using the EnVision System with diaminobenzidine (DAKO) according to the manufacturer's instructions. For immunocytochemistry of HepG2 cocultured with PBMC. After culture media were removed, the attached cells were fixed with methanol and then stained with mouse anti-human IDO1, followed by EnVision System with diaminobenzidine.

### Evaluation of immunohistochemical variables

Analysis was performed by two independent observers as described previously [39, 40]. At low-power field (×100), the tissue sections were screened and the five most representative areas were manually selected using an inverted research microscope (model DM IRB; Leica). For evaluating the proportion of IDO1-positive tumor cells, the respective areas were measured at high-power field (×600, ∼0.10 mm^2^/field). The number of the above cells was then counted manually.

### Immunoblotting

The proteins were extracted as previously described [32] and were separated by 10% SDS-PAGE, immunoblotted with an Ab against GAPDH (Abcam, Cambridge) or IDO1, and then visualized with an ECL kit (Pierce). For the coculture groups, PBMC were washed away before protein extraction. The expression of IDO1 protein in the attached cells was detected by western blot.

### Statistical analysis

Results are expressed as mean ± SEM unless otherwise indicated in the text. Statistical significance was determined by Student's *t* test. A value of *p< 0.05 was considered statistically significant.
